# Unconventional localization of electrons inside of a nematic electronic phase

**DOI:** 10.1073/pnas.2200405119

**Published:** 2022-10-18

**Authors:** Liam S. Farrar, Zachary Zajicek, Archie B. Morfoot, Matthew Bristow, Oliver S. Humphries, Amir A. Haghighirad, Alix McCollam, Simon J. Bending, Amalia I. Coldea

**Affiliations:** ^a^Centre for Nanoscience and Nanotechnology, Department of Physics, University of Bath, Bath BA2 7AY, United Kingdom;; ^b^Clarendon Laboratory, Department of Physics, University of Oxford, Oxford OX1 3PU, United Kingdom;; ^c^Institute for Quantum Materials and Technologies, Karlsruhe Institute of Technology, 76021 Karlsruhe, Germany;; ^d^High Field Magnet Laboratory, Radboud University, 6525 ED Nijmegen, Netherlands

**Keywords:** unconventional superconductors, magnetotransport, Fermi surface, quantum oscillations, thin flakes

## Abstract

The magnetotransport behavior inside the nematic phase of bulk FeSe reveals unusual multiband effects that cannot be reconciled with a simple two-band approximation proposed by surface-sensitive spectroscopic probes. In order to understand the role played by the multiband electronic structure and the degree of two-dimensionality, we have investigated the electronic properties of exfoliated flakes of FeSe by reducing their thickness. Based on magnetotransport and Hall resistivity measurements, we assess the mobility spectrum that suggests an unusual asymmetry between the mobilities of the electrons and holes, with the electron carriers becoming localized inside the nematic phase. Quantum oscillations in magnetic fields up to 38 T indicate the presence of a hole-like quasiparticle with a lighter effective mass and a quantum scattering time three times shorter, as compared with bulk FeSe. The observed localization of negative charge carriers by reducing dimensionality can be driven by orbitally dependent correlation effects, enhanced interband spin fluctuations, or a Lifshitz-like transition, which affect mainly the electron bands. The electronic localization leads to a fragile two-dimensional superconductivity in thin flakes of FeSe, in contrast to the two-dimensional high-$T_{\text{c}}$ induced with electron doping via dosing or using a suitable interface.

## Significance

Among iron-based superconductors, FeSe displays an anomalous electronic nematic state, strong electronic correlations, and orbitally dependent band shifts that can influence its superconducting pairing. Here, we report detailed magnetotransport studies of thin flakes of FeSe that reveal unconventional transport, in which the hole carriers remain highly mobile, whereas the mobility of the electron carriers is low, and weakly temperature dependent, inside the nematic phase. This suggests an unusual localization of negative charge carriers that may be caused by orbital-dependent enhanced correlations, scattering of spin fluctuations, and/or a topological electronic transition. As the superconductivity is suppressed by reducing the flake thickness, it suggests that the electron pockets participate actively in pairing. By doping, electron pockets expand, enabling high-Tc superconductivity.

Among different classes of unconventional superconductors, iron-based systems display rich physics due to their multiband structure and the competition between different electronic interactions (1). Iron-chalcogenides are among the most strongly correlated iron-based superconductors, and, due to the large intra-atomic exchange caused by the Hund’s coupling, the correlation strengths are expected to be strongly orbitally dependent (2). This orbital differentiation can lead to an orbital-selective Mott transition or spectral weight transfer, where the band with dominant *d_xy_* orbital appears more insulating, while other bands with *d_xz_* and *d_yz_* orbital character remain metallic (1, 3). As a result, electronic and superconducting properties are likely to be influenced by these effects, as seen in orbitally dependent band shifts in angle-resolved photoemission spectroscopy (ARPES) in iron-chalogenides (4, 5) and orbital-dependent Cooper pairing (6).

FeSe is a candidate system in which the presence of the lower-Hubbard band establishes the important role of electronic correlations, orbital-dependent band shifts in the nematic phase, and Fermi surface shrinking (7–10). The strength of these effects can be suppressed by isoelectronic substitution with sulfur (11–13). The interatomic Coulomb repulsion in FeSe can produce a strongly renormalized low-energy band structure, where the van Hove singularity sits remarkably close to the Fermi level in the high-temperature electron liquid phase (14). ARPES studies under strain suggest that in FeSe, one electron pocket either is missing or the spectral weight is transferred between its two electron pockets (3, 15). Furthermore, neutron scattering suggests the coexistence of both stripe and Nèel spin fluctuations with a substantial amount of spectral weight transferred toward stripe spin fluctuations inside the nematic phase (16). The lack of long-range antiferromagnetic order in FeSe has been linked to the competition between different types of magnetic order that can lead to significant magnetic frustration (17).

Transport properties of systems with orbitally dependent correlations are predicted to display a coherence–incoherence cross-over as a function of temperature (18), and, in addition, nematic iron-based superconductors are prone to anisotropic single-particle scattering enhanced by interband spin or charge fluctuations (19). In the presence of spin fluctuations, the scattering is strongly influenced by quasiparticles close to hot spots at Fermi surface locations where the nesting is strong (20, 21). Furthermore, the large fluctuating moments with Nèel and stripe magnetic instabilities (16) are likely to strongly affect the magnetotransport behavior. Magnetotransport studies of FeSe and FeSe${}_{1-x}$S*_x_* have identified a linear resistivity regime and a large magnetoresistance inside the nematic phase (22, 23). However, the low-field magnetotransport data in the normal state suggest that, in addition to one hole and one almost-compensated electron band, the nematic phase of FeSe exhibits an additional tiny electron pocket with a high mobility and nonlinear Hall coefficient (23, 24) that can also be found in the low-pressure regime (25). Additionally, the amplitude of the quantum oscillations at low temperatures and high magnetic fields indicates that hole carriers are likely to be more mobile than electron bands (23), and the highly mobile holes and enhanced spin fluctuations also dominate the high-$T_{\text{c}}$ pressure phase of FeSe (26).

In this paper, we present a detailed magnetotransport study of exfoliated thin flakes of FeSe, as compared with bulk single crystals. The mobility spectrum and a two-band model reveal that the mobility of the holes in thin-flake devices is much higher than that of electrons. Electrons become localized at low temperatures, due to the enhancement of the orbitally dependent correlations and enhanced anisotropic scattering in two-dimensional devices. From quantum oscillations, we find that the size of the extremal hole orbit is smaller, and the effective mass of the hole band is lighter than in bulk. In the low-temperature regime, the resistivity shows a linear dependence down to the lowest temperatures, but Fermi liquid behavior is restored in the cleanest flake, where quantum oscillations are present. While the thinner devices could be sensitive to increased impurity and surface scattering, the reduction in $T_{\text{c}}$ in thin flakes is directly correlated with the suppression of the nematic phase and the localization of electron carriers.

## Results

### Transport Properties of Thin Flakes of FeSe.

**Fig. 1.**
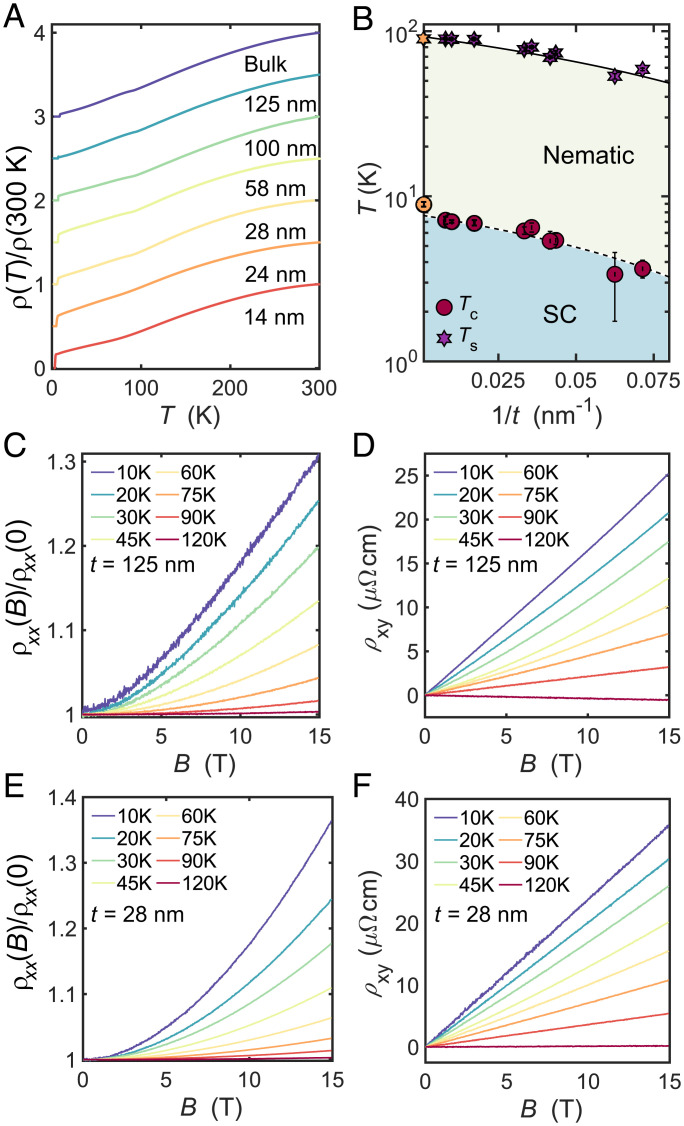
Magnetotransport of thin flakes of FeSe. (*A*) Temperature dependence of the normalized zero-field resistance *ρ*(*T*)/*ρ*(300 K) for a bulk crystal and different thin-flake devices (t=14 to 125 nm). Data are vertically offset, and this panel is updated with new data from ref. 27. (*B*) Inverse thickness *t* dependence of the superconducting transition temperature, $T_{\text{c}}$, and the structural transition temperature, $T_{\text{s}}$. Bulk values are shown on the left with light orange symbols. The dashed line is a fit to the Cooper model, and the solid line is a guide to the eye for the changes in $T_{\text{s}}$ with the thickness *t* (28, 29). (*C*–*F*) The magnetic field dependence of the longitudinal and transverse components of the resistivity, *ρ_xx_* and *ρ_xy_*, at different constant temperatures for two devices with t=125 and t=28 nm.

Fig. 1*A* shows the temperature dependence of the normalized zero-field resistance, *ρ*(*T*)/*ρ* (300 K), for a bulk crystal and six different thin-flake devices with thicknesses in the range t= 14 to 125 nm. Bulk FeSe undergoes a tetragonal to orthorhombic distortion at $T_{\text{s}}\approx 90$ K without any accompanying long-range magnetic order, followed by the onset of superconductivity at $T_{\text{c}}\sim 9$ K (30). These parameters in single crystals are sensitive to the growth conditions and the impurity level; the suppression of $T_{\text{c}}$ is affected by the increase in the amount of disorder, as measured by the residual resistivity ratio (*RRR*), which linearly correlates with the suppression of $T_{\text{s}}$ (31). In thin flakes, the superconducting transition temperature, $T_{\text{c}}$, is already lowered from the bulk single-crystal value to 7.2 K for a t=125-nm device, decreasing further to 3.6 K for a t=14 nm device, as reported previously (27). The suppression of superconductivity in the t=125-nm device occurs despite the high residual resistance ratio of RRR~32, which is similar to bulk crystals from the same batch (22). In thinner flakes, the *RRR* value falls as a function of decreasing thickness, reducing to 5.5 in the t=14-nm device, as shown in *SI Appendix*, Fig. S4*C*, being similar to the effect of impurity scattering by Cu doping in FeSe (32, 33). Similarly, the emergence of the nematic phase at $T_{\text{s}}$, which results in significant in-plane distortion of the Fermi surface and orbitally dependent band shifts (10), becomes smeared and less defined for thinner flakes. Fig. 1*B* shows the variation of $T_{\text{s}}$ and $T_{\text{c}}$ as a function of inverse thickness (1/t) for different devices, revealing that both are suppressed for thinner flakes; interestingly, we find a linear dependence between $T_{\text{s}}$ and $T_{\text{c}}$, as found in Cu-substituted FeSe (33) (*SI Appendix*, Fig. S4*B*). As these two parameters are correlated and are suppressed as the *RRR* ratio is reduced, it suggests that the two-dimensional confinement, enhanced fluctuations, and surface impurity scattering play an important role in the suppression of $T_{\text{c}}$ in thin flakes of FeSe (27).

### Low-Field Magnetotransport Behavior.

Fig. 1 *C*–*F* show the field dependence of the longitudinal magnetoresistance normalized by the zero-field value, *ρ_xx_*(*B*)/$\rho_{xx}(0)$, and the Hall resistivity, *ρ_xy_*, for two different devices with t= 125 and 28 nm, respectively. FeSe is a multiband stoichiometric compound, in which charge compensation requires that $n=n_{e}=n_{h}$. At high temperatures in the tetragonal phase, the magnetotransport properties of bulk FeSe can be accurately described by using a compensated two-band model, as detailed in *SI Appendix*. This model corresponds to a hole pocket at the Brillouin zone center and an electron pocket at the corner of the Brillouin zone (23). This two-band picture also describes the high-temperature magnetotransport behavior of thin flakes, whereby the magnetoresistance does not saturate and *ρ_xy_* shows a linear dependence on magnetic field (*SI Appendix*, Fig. S5).

**Fig. 2.**
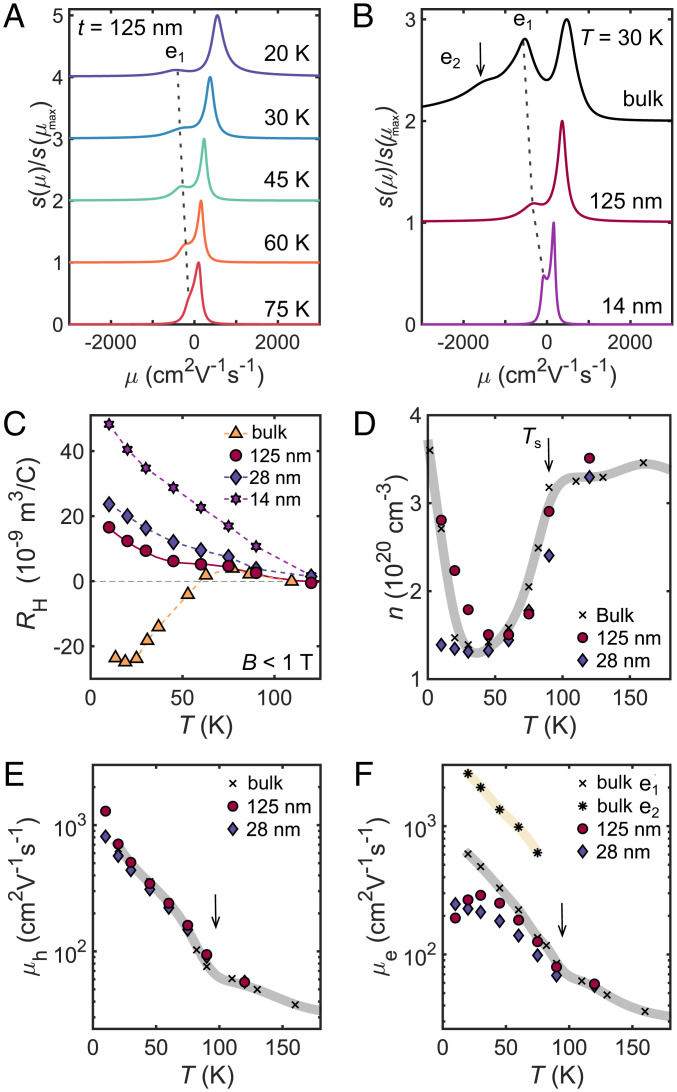
The changes in the carrier mobilities with temperature. (*A*) Temperature dependence of the spectra normalized to the maximum value $s(\mu_{\max})$ of electron-like (negative) and hole-like (positive) carriers for a t= 125-nm device. (*B*) A comparison of the normalized mobility spectra to the to the maximum value $s(\mu_{\max})$ for a bulk crystal and different thin-flake devices of FeSe at T=30 K. The spectra are vertically offset for clarity. The dotted line shows the mobilities of the electron-charge carrier *e*_1_, and the arrow indicates the highly mobile electron, *e*_2_, found only in the bulk. (*C*) The temperature dependence of the low-field Hall coefficient $R_{\text{H}}$ (extracted for *B*  <  1 T) for a bulk FeSe crystal and three thin-flake devices. (*D*) Temperature dependence of the carrier density extracted by using a compensated two-carrier model $n=n_{e}=n_{h}$ (*SI Appendix*). (*E* and *F*) Temperature dependence of carrier mobilities for the hole-like carriers, *μ_h_* (*E*), and electron-like carriers, *μ_e_* (*F*), for various samples. The data points for the bulk single crystals of FeSe and the three-carrier model parameters are from ref. 23, and the solid lines are guides to the eye. The vertical arrows indicate the position of $T_{\text{s}}$.

Inside the nematic phase (below 75 K), the Hall component *ρ_xy_* of bulk crystals of FeSe displays nonlinear behavior in magnetic field and a negative slope (23) as well as a deviation from the compensated two-band behavior, whereas all thin-flake devices display a positive Hall component in magnetic field (see also *SI Appendix*, Fig. S5). To visualize the differences between the bulk and thin flakes, Fig. 2*C* shows the temperature-dependence of the Hall coefficient, $R_{\text{H}}=\rho_{xy}/B$, in low magnetic fields (*B*  <  1 T). All the thin-flake devices have a positive Hall coefficient below $T_{\text{s}}$, suggesting that the transport behavior becomes increasingly dominated by the hole-like carriers. We detect a local maximum in $R_{\text{H}}$ around 65 K for t=125 nm, which is close to the temperature at which the Hall coefficient of bulk FeSe (23) and also the resistivity anisotropy under strain change sign (34). These striking changes in transport behavior could signify the development of anisotropic scattering effects inside the nematic phase, which can be enhanced by lowering the temperature and reducing the thickness. The $\rho_{xy}(B)$ component deviates from a linear dependence in magnetic field in the same temperature regime (see corresponding derivatives in *SI Appendix*, Fig. S5). However, linear dependence is detected at the lowest temperatures, in the regime of isotropic scattering caused mainly by impurities, and at the highest temperatures, where electron–phonon scattering becomes important, similar to other reports on thin flakes (35). Overall, the behavior of the Hall coefficient in thin flakes of FeSe is in stark contrast to bulk FeSe, in which $R_{\text{H}}$ becomes negative below the nematic transition, as seen in Fig. 2*C*.

### Mobility Spectrum.

To analyze in detail the origin of the changes inside the nematic phase, we perform a mobility-spectrum analysis of the magnetotransport data (Fig. 2). This approach has previously been successful in characterizing the transport behavior of bulk FeSe (24) and other multiband systems (36). The modeling is based on the methodology detailed in refs. 37 and 38, which eliminates the need for making a priori assumptions on the transport parameters in a multicarrier system and describes the mobility spectrum, s(μ)=eμn(μ), with the negative electron charge assigned to a negative value of mobility. Fig. 2*A* shows the evolution of the normalized mobility spectrum *s*(*μ*)/*s*(*μ_max_*) for the t=125-nm device as a function of temperature. The height of *s*(*μ*) could be linked to the variation of the number of carriers, n(μ), whereas the width of the peak of s(μ) relates to a distribution of relaxation times and the magnetic field resolution, such that μB<1 (37) (*SI Appendix*, Fig. S9). As a function of temperature, the mobility of positive charge carriers increases strongly with decreasing temperature reaching $\mu_{h}\sim 1,200$ cm^2^ ⋅V^-1^ ⋅s^-1^ at 10 K. On the other hand, the electron mobility has a much weaker increase with decreasing temperature, and below 50 K, the corresponding mobility peak position only shifts slightly from $\left|\mu_{e1}|\sim 300(50\right)$ cm^2^ ⋅V^-1^ ⋅s^-1^. As the mobility of the holes is larger than that of electrons, it explains the positive sign of the Hall coefficient in thin flakes, shown in Fig. 2*C*.

Next, we compare the mobility spectrum of two different flake devices with those of the bulk crystal at 30 K, as shown in Fig. 2*B*. The bulk mobility spectrum has a complex shape for the electron-like charge carriers, which could indicate the presence of an additional highly mobile electron band, *e*_2_ (or the existence of sharp changes in curvature of the Fermi surface for electron-like flower shape pockets, *see* Fig. 4*B*), besides the mobility peak corresponding to the high-carrier-density electron band, *e*_1_. This behavior is in agreement with a previous report indicating a broad mobility spectrum for electrons, with the highly mobile carriers assigned as ultra-fast Dirac-like carriers in bulk FeSe (24). This highly mobile carrier, *e*_2_, is not visible in any of the thin flakes, even for the 125-nm flake which has a similar value of *RRR* to that of the bulk. This suggests a high sensitivity of the electronic structure, in particular the electron pockets, to reduced interlayer coupling and enhanced two-dimensional confinement. Fig. 2*B* also shows that the mobilities of both electron and hole carriers, in general, decrease with the thickness of the flakes and reduced *RRR* ratio (*SI Appendix*, Fig. S4*C*). In the case of the thinnest flake with t=14 nm, both the electron *e*_1_ and hole mobilities have been drastically reduced, as compared to the bulk crystal ($|\mu_{e1}|=80$ and $\mu_{h}=160$ cm^2^ ⋅V^-1^ ⋅s^-1^ at 30 K).

To provide quantitative insights into the behavior of the charge carriers in the thin flakes, we simultaneously fit the two resistivity components, *ρ_xx_* and *ρ_xy_*, to the compensated two-band model using the values from the mobility spectrum as starting parameters. Fig. 2 *D*–*F* show the temperature dependence of the carrier density, $n=n_{h}=n_{e}$, and the field-independent mobilities, *μ_h_* and *μ_e_*, compared with those for the bulk single crystals of FeSe (23). At high temperatures, the extracted values for *n* are similar to those of bulk samples, which show a relatively constant carrier density of $n\approx 3-4\times 10^{20}$ cm^-3^. Unexpectedly, the carrier density shows a marked reduction of more than a factor of 2 at 45 K, before rapidly increasing back to the high-temperature value below 10 K. This drop in carrier density below $T_{\text{s}}$ cannot be reconciled with the expectation that the Fermi surface pockets should only deform, but not change size, inside the nematic phase, in the absence of any spin-density wave (SDW) order. This anomalous behavior may be caused by the onset of strongly anisotropic scattering at the Fermi surfaces below $T_{\text{s}}$ (11), arising from the presence of strong spin fluctuations (39), as different parts of the Fermi surfaces that are nested by the antiferromagnetic ordering vector will experience a dramatic increase in the scattering rate (40). The calculated drop in the effective carrier density, *n*, in the absence of any change in the Fermi surface volume could be a manifestation of strongly anisotropic scattering inside the nematic phase or that some of the charge carriers become more localized and do not contribute to transport behavior. However, *n* recovers its value in the low temperature limit for the t=125-nm flake, where the isotropic impurity scattering dominates, similar to the bulk behavior (23).

Fig. 2 *E* and *F* show the extracted mobilities from the two-band analysis, which confirms the striking difference in the mobility behavior of the two types of charge carriers (*μ_h_* and $\mu_{e1}$). For all the measured thin-flake devices, the hole mobilities are much larger than those of electrons, exhibiting a similar temperature dependence to that of the bulk band. There is a slightly decreased mobility of $\mu_{h}=810$ cm^2^ ⋅V^-1^ ⋅s^-1^ at T=10 K for the t=28-nm sample, attributed to the increasing importance of surface scattering due to the increasing surface-to-volume ratio in thinner samples, as well as the reduction of the residual resistivity ratio due to other extrinsic effects (additional scattering from charged centers in the SiO_2_ substrate). In contrast to the behavior of the holes, the electron mobility of thin flakes displays a much weaker temperature dependence that deviates significantly from the bulk *e*_1_ value. Actually, the electron mobility plateaus in thin-flake samples at low temperatures, as is clearly seen in the mobility data of Fig. 2*F*. The contrasting behavior of the mobilities of the holes and electrons is unexpected and correlates with the suppression of superconductivity in thin flakes in two dimensions. This behavior in FeSe is different from that found in iron-pnictides, where electrons are often more mobile than holes (41). Our findings are in broad agreement with the results of terahertz spectroscopy in FeSe thin films that detect that the scattering time of the hole carrier becomes substantially longer than that of the electron at lower temperatures (42). Highly mobile hole carriers were also found in the high-$T_{\text{c}}$ phase of bulk FeSe under pressure (43).

### High-Field Magnetotransport.

**Fig. 3.**
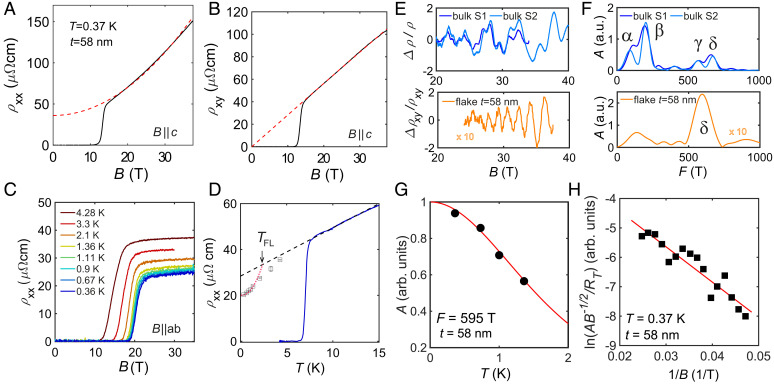
High-field magnetotransport and quantum oscillations in a thin-flake device of FeSe. (*A* and *B*) Longitudinal resistivity (*A*) and Hall resistivity (*B*) as a function of *B* for a t=58-nm flake at T=0.37 K. The dashed lines are fits to a two-band model with $\mu_{h}=1,017$ cm^2^ ⋅V^-1^ ⋅s^-1^ and $\mu_{e}=231$ cm^2^ ⋅V^-1^ ⋅s^-1^. (*C*) In-plane resistivity as a function of magnetic field at different fixed temperatures. (*D*) Low temperature zero-field resistivity together with extrapolated data from *C* assuming that *ρ_xx_* is linearly dependent on *B* in *C* for H||(*ab*) (open squares). The dashed line is a linear fit to the data. The arrow indicates the cross-over to a Fermi liquid *T*^2^ behavior below $T_{\text{FL}}$ (dotted line). (*E*) Quantum oscillations, $\Delta \rho_{xy}/\rho_{xy}$, of the t=58-nm flake and two bulk single crystals in *ρ_xy_* for S1 and *ρ_xx_* for S2. (*F*) The corresponding FFT spectra from *E* for the flake and the bulk sample. The amplitude of the oscillations of the t=58-nm flake is multiplied by a factor of 10 in *E* and *F* in arbitrary units. (*G*) The temperature dependence of the oscillation amplitude for the t=58-nm flake. A Lifshitz–Kosevich fit (solid line) yields an effective carrier mass of $m_{\text{eff}}=3.1(2)$m${}_{\text{e}}$ for the hole pocket *δ*, located at the center of the Brillouin zone. (*H*) The corresponding Dingle plot for the estimation of the slope, which gives $T_{\text{D}}=4.5(4)$ K.

Fig. 3 *A* and *B* show the magnetotransport behavior of a t=58-nm flake in high magnetic fields up to 37.5 T. The Hall resistivity is observed to be strictly linear in magnetic field, as expected for a perfectly compensated two-band system. However, the longitudinal magnetoresistance exhibits an unconventional $B^{1.6}$ dependence for different devices (*SI Appendix*, Figs. S6 and S7), similar to that found in bulk FeSe at high magnetic fields (22). Fig. 3*C* shows in-plane magnetotransport studies that are not affected by orbital effects, as the current and magnetic field are parallel to each other; the linear high-field extrapolation is used to access the low-temperature normal resistivity, as shown in Fig. 3*D*. We find that the low-temperature resistivity has a linear temperature dependence to the lowest temperatures for most of the measured flakes (*SI Appendix*, Figs. S7*F* and S8*B*), except for the one that displays quantum oscillations, as shown in Fig. 3*D*. A cross-over transition to the Fermi liquid behavior occurs below 5 K for t=58 nm, but this cross-over is highly sensitive to the degree of impurity scattering, as found in FeSe${}_{1-x}$S*_x_* (22) and Cu-substituted FeSe (33). Linear dependence at the lowest temperature is found for a flake with t= 100 nm (*SI Appendix*, Fig. S7*F*), and it describes the resistivity behavior below 50 K for both orthorhombic directions in another flake in *SI Appendix*, Fig. S8. This behavior is often a hallmark of scattering by spin fluctuations in the vicinity of an antiferromagnetic critical point (44, 45).

**Fig. 4.**
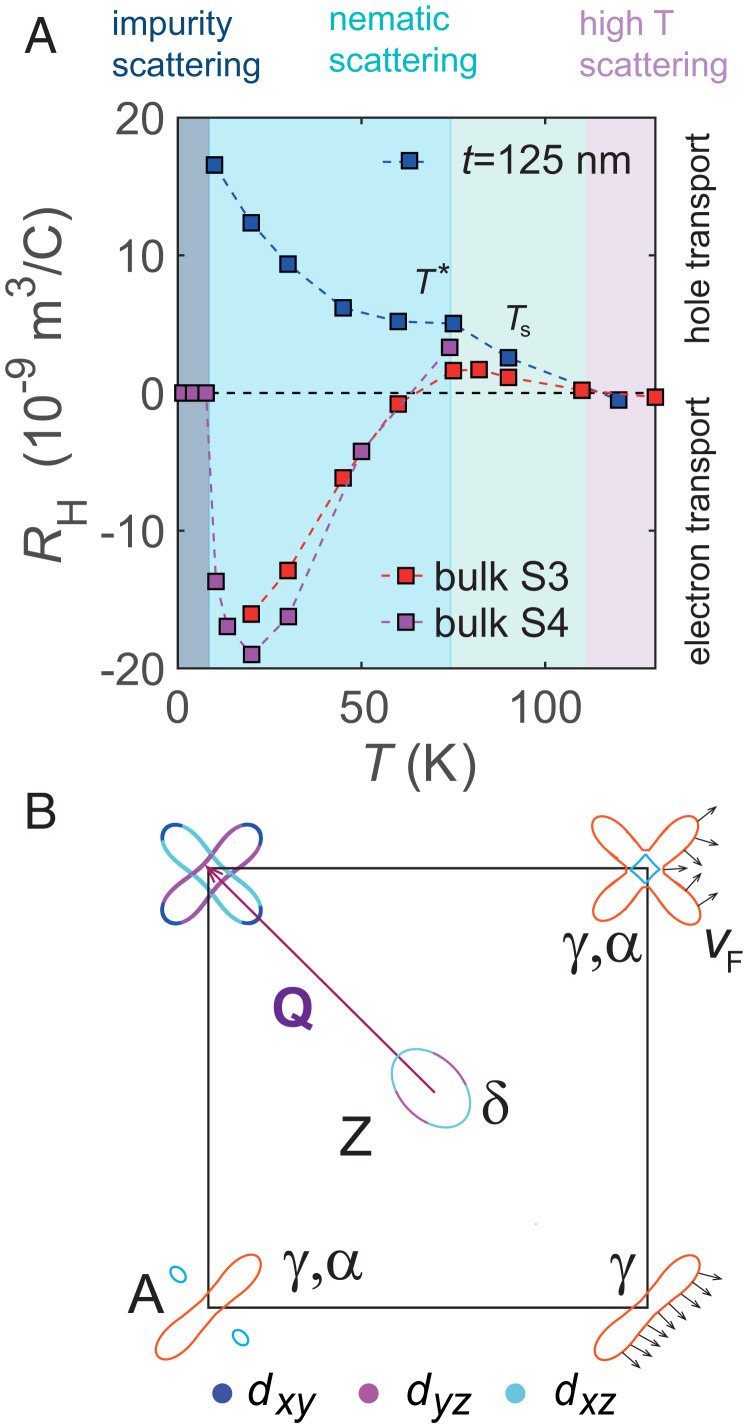
Anomalous transport of FeSe. (*A*) The temperature dependence of the Hall coefficient in bulk single crystals (S3 from ref. 23 and S4 from ref. 22), as compared with a clean t=125-nm flake of FeSe, with similar RRR~32 value (*SI Appendix*, Fig. S4*C*). The potential different regimes of scattering are illustrated by different colored regions, as detailed in ref. 40. Inside the nematic phase, the orbital ordering and spin–orbit coupling can generate sizable in-plane anisotropy in susceptibility below $T^{*}$ (47). (*B*) Schematic Fermi surface of FeSe at low temperatures based on quantum oscillations for $k_{z}=\pi /c$ (13, 48). The electron orbits in the corner of the Brillouin zone reflect different representations induced by orbital ordering, spin-orbit coupling, and strain (13, 49–53) from two electron pockets (top corners) to a single peanut pocket (bottom right) or a single peanut with two small pockets (bottom left). The small arrows indicate the variation of the Fermi velocity when the magnetic field is normal to the plane of the pocket that could generate electron and hole contributions to the Hall effect (54). The dominant orbital character of the Fermi surface is shown in the top left corner. Hot spots can be generated at the crossing point between elliptical electron pockets (40).

At the lowest temperatures, we have detected quantum oscillations for one of the flakes with t=58 nm, both in *ρ_xx_* and *ρ_xy_* components (Fig. 3 *A* and *B*), with the amplitude of the signal in the Hall component being stronger. Fig. 3*E* shows quantum oscillations in the *ρ_xy_* component having an amplitude a factor of 10 smaller than bulk single crystals (23). The fast Fourier transform (FFT) spectra help to identify the extremal areas of the Fermi surface pockets normal to the applied magnetic field, *A_ki_*, via the Onsager relation, $F_{i}=A_{ki}\hbar /(2\pi e)$ (46). The low-temperature experimental Fermi surface of FeSe is composed of one warped cylindrical hole band at Γ with oscillation frequencies *β* (*k_z_*  =  0, F=164 T) and *δ* ($k_{z}=\pi /c$, F=664 T) and potentially two warped cylindrical electron Fermi surfaces that are located at the corners of the Brillouin zone (Figs. 3*F* and 4*B*) (7, 10, 46). Fig. 3*F* shows that the dominant oscillation frequency of the t=58-nm thin flake is 595 T, which is likely to correspond to the largest orbit at the *Z* point of the hole band *δ*. The signal from the hole bands was also found to be dominant in the *ρ_xy_* for bulk crystals (23). The observed reduction in the size of the extremal area of the Fermi surface of the thin flake could suggest a reduction of *k_z_* warping due to an increase in the degree of two-dimensionality in the thin flakes. The cyclotron-averaged effective masses of the quasiparticles extracted from the temperature dependence of the amplitude of the quantum oscillations in Fig. 3*G* (using raw data from *SI Appendix*, Fig. S6*A*) was found to be $\sim 3.1(2)\;m_{e}$, slightly lighter than $\sim 4.5(1)\;m_{e}$, found for the bulk *δ* pocket (7, 53).

### Scattering.

In order to quantify how the amplitude of quantum oscillations is affected by impurity scattering, we estimate the Dingle temperature, $T_{\text{D}}$, as shown in Fig. 3*H* and detailed in *SI Appendix* and ref. 54. The slope gives a $T_{\text{D}}=4.5(4)$ K, which corresponds to a quantum mean free path of $\ell_{q}\sim 140$ Å for the t=58-nm device. The quantum scattering time, $\tau_{q}=\hbar$/($2\pi k_{B}T_{\text{D}}$), corresponds to the time taken to fully randomize the linear momentum of an electron; it is found to be $\tau_{q}=0.27(2)$ ps, corresponding to a quantum hole mobility of $\mu_{q}\sim 158$ cm^2^/Vs. This quantum scattering time for the *δ* hole pocket is almost a factor of 3 shorter than in bulk FeSe, where $\tau_{q}=0.7(1)$ ps, as shown in *SI Appendix*, Fig. S2. This could indicate an increase in the impurity and surface scattering in this thin flake, as its *RRR* is smaller than the bulk (Fig. 4*B*). Furthermore, the classical mobility from the two-band model yields a value close to $\mu_{h}=1,017$ cm^2^ V ⋅^-1^ ⋅s^-1^, corresponding to a classical scattering time of $\tau_{h}\sim 1.8$ ps, using the effective mass from quantum oscillations of 3.1(2) m*_e_*. The large difference (a factor of 6) between the two types of scattering processes occurs due to the sensitivity of the quantum mobility to both small- and large-angle scattering events, while the transport mobility is mainly dominated by large-angle scattering (55).

## Discussion

The unconventional behavior in magnetotransport of thin flakes of FeSe suggests the strong sensitivity of the quasiparticle scattering and Fermi surface inside the nematic phase (Fig. 4*A*). Below $T_{\text{s}}$, there are significant changes in the shape of the Fermi surface caused by orbitally band-dependent shifts (10, 13). The three-dimensional hole pocket, centered at the Z point, is pushed below the Fermi level due to orbital ordering, and the in-plane pockets become strongly elongated (Fig. 4*A*). This change of the in-plane anisotropy is not expected to change the carrier density, *n*, by a factor of 2 inside the nematic phase (10, 13), which normally occurs when the Fermi surface undergoes a significant reconstruction, as for BaFe_2_ As_2_ in the presence of the SDW phase (56). Thus, the drastic changes in the Hall coefficient and carrier densities of FeSe imply either that certain charge carriers inside the nematic phase scatter much more or get redistributed onto more localized *d_xy_* bands, and, thus, do not participate in conduction. Additionally, the small quasi-two-dimensional Fermi surface pockets could also suffer topological changes inside the nematic phase (Fig. 4*B*), as found in thin films of FeSe (51) or induced by small applied strain (49).

The Hall coefficient, in the low field limit, is very sensitive to the momentum-dependent scattering and the curvature of the Fermi surface. The Fermi surface topology and the changes in curvature from convex to concave around a pocket lead to the variation of the scattering-path vector, $l_{k}=v_{F}\tau_{k}$. The enclosed area swept by the $l_{k}$ vector, as **k** moves around the Fermi surface, will change sign, which directly affects the sign of the Hall conductivity (52) (*SI Appendix*, Fig. S10). The Fermi surface of FeSe at low temperatures has an elliptical hole pocket and one or two electron pockets, as represented in Fig. 4*B*. The difference between the Hall coefficient of bulk and thin flakes in Fig. 4*A* could be linked to a topological change of the Fermi surface, induced by orbital ordering (13); this could transform a flower-shape pocket, with convex and concave curvatures and negative Hall coefficient, into an elongated ellipse in thin flakes, with a convex curvature that gives a positive Hall coefficient (Fig. 4*B* and *SI Appendix*, Fig. S10). The small electron pocket *e*_2_ of the bulk (*α* pocket) is absent in the mobility spectrum of thin flakes of FeSe. As the inner electron band is located very close to the Fermi level, it is highly sensitive to small changes in energy (~3 meV), upon reducing the thickness of the flakes and under applied uniaxial strain. This pocket already disappears in thin films of FeSe (51) (Fig. 4*B*), due to the orbital and momentum-dependent energy splitting at the M point that is larger for thin films (~70 meV) (51) than in bulk (~50 meV) (10). Furthermore, ARPES studies of bulk FeSe under strain, which probe the surface and bulk layers up to ~10 Å (57), usually detect a single electron pocket in the corner of the Brillouin zone that can be induced by applied strain (49, 50) (Fig. 4*B*).

The strong disparity between the hole and electron mobilities (up to a factor of 6 at 10 K) and the observation of the rather temperature-independent mobility of the electron carriers below 50 K could imply an enhancement of the orbitally averaged effective masses and/or orbitally dependent scattering that affect mainly the electron pockets. The proximity to a Van Hove singularity caused by orbitally dependent shifts in FeSe can also amplify the small-angle scattering processes for the electron pockets. Using the low-temperature mobility values of electrons of *μ_e_* = 231 cm^2^/(Vs) and τ~1.8 ps from the two-band model (Fig. 3 *A* and *B*), one can estimate the effective mass to be $\sim 13\;m_{\text{e}}$ for the t=58-nm flake. This value is much larger than the orbitally averaged effective mass of ~7 m*_e_* of the electron pocket *γ* for bulk crystals (Fig. 4*B*) (7), and it would be difficult to detect experimentally in quantum oscillations (Fig. 3*F*). Assuming that the effective masses of heavy electrons are the same for the bulk and thin flakes, then the changes in mobilities between electron and hole carriers could reflect an anisotropy in scattering (classical scattering time being ~0.9 ps for electrons and 1.8 ps for holes). Interestingly, the effective mass extracted from ARPES studies is much smaller, $\sim 1m_{\text{e}}$, for a single momentum direction (7, 13), and it can be enhanced in thin films and flakes of FeSe up to $\sim 4m_{\text{e}}$, both using K-dosing of FeSe (58) and ionic liquid gating (59). Orbitally dependent band shifts and renormalizations were detected previously in FeSe, with the dominant *d_xy_* hole band being the most renormalized by a factor of 8, as compared with a factor 2.5 to 3.5 for the *d_xz_* and *d_yz_* orbitals (2, 7). Interestingly, the part of the electron pockets with *d_xy_* character can become completely incoherent (Fig. 4*B*) and hard to detect in surface-sensitive experiments (60). Alternatively, there is an exchange of *d_xy_* spectral weight from one electron pocket toward the other electron pocket, as suggested by recent ARPES studies under strain (3). Furthermore, the orbitally dependent pairing between electron and hole pockets was detected from surface studies using scanning tunneling microscopy measurements of FeSe (60, 61).

The Hall coefficient $R_{\text{H}}$ of FeSe increases with decreasing temperature and has an inflection point at $T^{*}\sim 75$ K, for both bulk and thin flakes, as shown in Fig. 4*A*. On cooling, $R_{\text{H}}$ starts deviating, and *n* is significantly reduced inside the nematic phase, as if there is a loss of available charge carriers (Fig. 2*D*). At the temperature $T^{*}$, applied strain has the weakest effect on resistivity and the transport anisotropy changes sign (34). The change in resistivity anisotropy coincides also with the temperature at which a large anisotropy develops in the local spin susceptibility (62). Thus, the anisotropy of the local magnetism affects the quasiparticle scattering and the coherent coupling between local spins and itinerant electrons. The sign of the Hall coefficient is always positive in thin flakes, but negative in the bulk below ~60 K, despite having similar *RRR* values. The Hall coefficient is also positive in Cu-substituted FeSe with large impurity scattering (32, 33) and in thin films of FeSe${}_{1-x}$S*_x_* (63). Furthermore, it also becomes positive by using the isoelectronic sulfur substitution in single crystals of FeSe${}_{1-x}$S*_x_* (for *x*  >  0.11) (22, 64, 65), due to subtle changes in the band structure and spin-fluctuation scattering (13, 22). At the lowest temperature, there is a cross-over from inelastic to impurity-dominated scattering, when the *n* value normally recovers to the high-temperature tetragonal case (Fig. 4*A*). The very large increase in $R_{\text{H}}$ observed in the thinnest t=14-nm flake resembles the behavior of thin films of FeSe with lower *RRR* values (59). Recently, it was shown theoretically that the impurity scattering in FeSe can give rise to anisotropic scattering and anisotropy in resistivity (66). Furthermore, a strong role of orbital differentiation on the temperature dependence of $R_{\text{H}}$ has also been found in other systems, like Sr_2_ RuO_4_ (67) and FeCrAs (68).

Despite the lack of long-range magnetic order in FeSe, there is a large energy range of magnetic fluctuations due to the relatively small spin-fluctuation bandwidth together with the low carrier density (16). In zero-magnetic field in thin flakes, we detect a linear resistivity below 50 K in most flakes (*SI Appendix*, Fig. S8*B*), except in the cleanest samples, in which a cross-over to Fermi liquid behavior occurs (Fig. 3*D*). The linear resistivity occurs for both orthorhombic directions, consistent with scattering by critical antiferromagnetic fluctuations in the presence of disorder, which is strongly enhanced at hot spots on the Fermi surface, where the nesting is perfect (Fig. 4*B*) (19, 21, 44). In the presence of spin fluctuations, the quasiparticle currents dressed by vertex corrections acquire the character of the majority carriers and lead to a larger absolute Hall coefficient with a marked temperature dependence (69). Additionally, the localization of electrons could be enhanced by spin fluctuations that affect interband scattering between elliptical electron pockets, like the Nèel-type fluctuations, as compared with interband stripe order fluctuations between holes and electrons (Fig. 4*B*). Short-range, weak Nèel fluctuations strongly suppress the $s_{\pm }$ superconducting state and can lead to a low-$T_{\text{c}}$ *d*-wave state (70).

## Concluding Remarks

In summary, we have performed a detailed study of electronic transport of high-quality FeSe thin flakes and identify an unusual localization effect of negative charge carriers inside the nematic phase. This disparity between hole and electrons emphasizes the anomalous transport inside the nematic phase, driven by the subtle interplay between the changes in the electronic structure of a multiband system and the unusual scattering processes induced by orbital-dependent enhanced correlations and/or anisotropic spin fluctuations. The two-dimensional confinement of thin flakes affects the mobility of the electron-like carriers significantly, but also plays a role in their superconductivity, which is suppressed. These effects emphasize the complexity and sensitivity of the electron pockets in FeSe-based systems, which are involved in the stabilization of a two-dimensional high-$T_{\text{c}}$ superconductivity via electron doping induced by interfacial effects or dosing.

## Materials and Methods

### Experimental Details.

Thin FeSe flakes were mechanically exfoliated from high-quality single crystals onto silicone elastomer polydimethylsiloxane stamps. Flakes of suitable geometry and thickness were then transferred onto Si/SiO_2_ (300-nm oxide) substrates with prepatterned Au-contacts using a dry transfer set-up housed in a nitrogen glovebox with an oxygen and moisture content <1 part per million. To minimize environmental exposure, a capping layer of thin (~20 nm) hexagonal boron nitride was transferred on top of the FeSe flake. The thickness of each sample was measured by using an atomic force microscope after all magnetotransport measurements had been performed. Magnetotransport measurements at temperatures down to 2 K and magnetic fields up to 16 T were performed by using a Quantum Design Physical Property Measurement system in Oxford, with high field measurements performed at the High Field Magnet Laboratory in Nijmegen (up to 37.5 T) in a Helium-3 cryostat. The magnetoresistance and Hall-resistivity contributions were separated by symmetrizing and antisymmetrizing the data obtained in positive and negative magnetic fields. The nonideal flake and contact geometries were accounted for by numerically evaluating the resistance to resistivity conversion factors. Details of these calculations are provided in *SI Appendix*, Fig. S1.

## Supplementary Material

Supplementary File

## Data Availability

The data that support the findings of this study are available through the open access data archive at the University of Oxford (ORA) (https://doi.org/10.5287/bodleian:X5GgyEj1O) (71). The data will be available upon the publication of the paper to the link above. Previously published resistivity data in zero magnetic field used on this work were part of the initial characterization of the thin flake devices of FeSe. Some of the resistivity curves in Fig. 1*A* and points in Fig. 1*B* were part of a figure dedicated to the study of the superconducting properties of thin flakes of FeSe (figure 1 in ref. 27). For the purpose of Open Access, the author has applied a CC BY public copyright license to any Author Accepted Manuscript version arising from this submission.
