# Supplementary material for: Unconventional localization of electrons inside of a nematic electronic phase
Source: Proc Natl Acad Sci U S A. 2022 Oct 18;119(43):e2200405119. doi: 10.1073/pnas.2200405119 (PMC9618067; doi:10.1073/pnas.2200405119)
Supplement: Supplementary File [file pnas.2200405119.sapp.pdf]

Supplemental Information: Unconventional localization of electrons inside a nematic electronic phase

Geometrical corrections

The exfoliation of rectangular-shaped flakes onto pre-patterned contacts leads to samples with rather non-ideal geometries. The current flow is inevitably quite inhomogeneous and the contacts extend a long way underneath the flake, violating the assumptions of the van der Pauw approach commonly used for irregularly-shaped samples. As a consequence the longitudinal and Hall resistivities have been estimated from finite difference solutions of the transport equation for a realistic sample and contact geometry.

For our two-dimensional problem the equation to be solved is given by:

$$\vec{E} + \rho_{xy}\vec{J} \times \hat{z} = \sigma_{xx}^{-1}\vec{J}, \tag{S1}$$

where $\vec{E}$ is the 2D electric field and $\vec{J}$ the 2D current density, $\hat{z}$ is unit vector perpendicular to the sample and is the direction of the applied magnetic field, $\vec{B}$=$\mu_0\vec{H}$; σ_{xx} and $\rho_{xy} = (B)/(n_{2D}e)$ are the sheet conductivity and Hall resistivity of the flake respectively, with n_{2D} the carrier concentration.

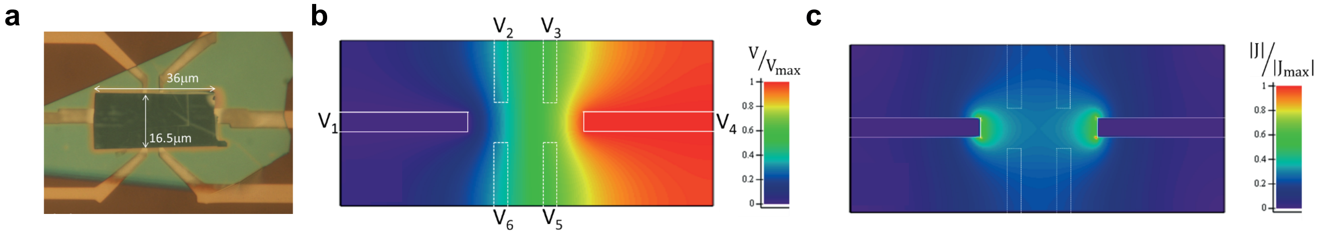

FIG. S1. **Simulation of the current and voltage distribution in a thin flake device.** Optical image of a FeSe flake device with $t = 125$ nm. (b) Calculated potential distribution, V, and (c) the modulus of the calculated current density, J, for a 144 × 66 site model of the sample geometry shown in (a) with $g = \sigma_{xx}\rho_{xy}$ = 0.01. Superimposed solid and dotted lines indicate the boundaries of the underlying current and voltage leads, respectively.

Fig. S1 illustrates an example used to solve Equation S1 in terms of the potential, V, on a 144 × 66 site grid. We impose the following boundary conditions along the left and right edges of the flakes where $J_x = 0$, the two components of the electric field satisfy $E_x = -(\sigma_{xx}\rho_{xy})E_y$, whereas along the top and bottom edges where $J_y = 0$, the two components of the electric field satisfy $E_y = (\sigma_{xx}\rho_{xy})E_x$. Once the solution has converged the current passing through the device, I, is calculated by summing the following expression for J_x down a vertical line through the centre of the device.

$$J_x = \frac{\sigma\left(E_x + (\sigma_{xx}\rho_{xy})\,E_y\right)}{\left(1 + (\sigma_{xx}\rho_{xy})^2\right)} \tag{S2}$$

We assume that the current leads are strongly coupled to the sample and arbitrarily fix $V_1 = 0V$ and $V_4 = 1V$ everywhere in the flake directly above them. In contrast we assume that the voltage contacts are only weakly coupled to the flake reflecting the strong anisotropy of our layered materials (a factor larger than 4 which increases with the reduction in the flake thickness [1]), and calculate the potential at each of the voltage leads as the average of the potential in the flake directly above it. It is then straightforward to show that the scaling factors (which are typically in the range 1-3) that must be applied to obtain the longitudinal resistivity, $\rho_{xx} = 1/\sigma_{xx}$, and the Hall resistivity, ρ_{xy}, from the experimentally-measured longitudinal resistance, R_{xx}, and Hall resistance, R_{xy}, are:

$$\begin{aligned} \rho_{xx} &= \left(\frac{I}{V_3 - V_2}\right) \times R_{xx}, \\ \rho_{xy} &= \left(\frac{I \cdot g}{V_2 - V_6}\right) \times R_{xy} \end{aligned} \tag{S3}$$

Here $g = \sigma_{xx}\rho_{xy}$ =0.01 is a parameter that is used in the numerical calculation to effectively define the magnetic field used to solve for the Hall voltage. Using the definitions in Equations S3 then the conversion factors for each resistivity component are $G_{xx} = I/(V_3 - V_2)$ and $G_{xy} = I \cdot g/(V_2 - V_6)$. Since Equation S1 yields a Hall voltage that is linear in magnetic field, H, the scaling factor for the Hall resistivity calculated in Equation S3 does not depend on this.

Two-band model

Considering a multi-band system in which current is applied along the x in-plane axis of a sample with a magnetic field applied along the z out-of-plane axis, the total resistivity is given by $\rho = \left(\sum_i \rho_i^{-1}\right)^{-1}$ assuming parallel network resistor which in an applied magnetic field B leads to:

$$\rho_i = \begin{pmatrix} \frac{1}{\sigma_i} & -R_i B \\ R_i B & \frac{1}{\sigma_i} \end{pmatrix} \tag{S4}$$

where the conductivity $\sigma_i = |n_i e \mu_i|$ and the Hall coefficient $R_i = -1/n_i e$ contain the carrier densities n_i and mobilities μ_i of i number of bands.

$$\rho_i = \frac{1}{|n_i e \mu_i|} \begin{pmatrix} 1 & -\mu_i B \\ \mu_i B & 1 \end{pmatrix} \tag{S5}$$

For a two-carrier system, the different components of the resistivity tensor are given by:

$$\rho_{xx} = \frac{(\sigma_1 + \sigma_2) + \sigma_1\sigma_2\left(\sigma_1 R_1^2 + \sigma_2 R_2^2\right) B^2}{(\sigma_1 + \sigma_2)^2 + \sigma_2^2\sigma_2^2 (R_1 + R_2)^2 B^2} \tag{S6}$$

and

$$\rho_{xy} = B\frac{\left(\sigma_1^2 R_1 + \sigma_2^2 R_2\right) + \sigma_1^2\sigma_2^2 R_1 R_2 (R_1 + R_2) B^2}{(\sigma_1 + \sigma_2)^2 + \sigma_1^2\sigma_2^2 (R_1 + R_2)^2 B^2} \tag{S7}$$

FeSe in the tetragonal phase can be described as a compensated two-band system, and we assign the conductivities to correspond to a hole σ_h and electron band σ_e, respectively. Compensation requires that R_1 = -R_2 and $n = n_e = n_h$ leading to a simplified form of the expression above

$$\rho_{xx} = \frac{1 + \frac{1}{(ne)^2}\sigma_h\sigma_e B^2}{(\sigma_h + \sigma_e)} \tag{S8}$$

and

$$\rho_{xy} = \frac{\frac{1}{(ne)}(\sigma_h - \sigma_e) B}{(\sigma_h + \sigma_e)}. \tag{S9}$$

By fitting simultaneously the magnetic field dependence of both the longitudinal magnetoresistance, ρ_{xx}, and the transverse Hall component, ρ_{xy}, to the above equations, the carrier density n and the two mobilities μ_e and μ_h can be extracted.

The mobility spectrum

The mobility spectrum has been developed to eliminate the need for making a priori assumptions on the transport parameters in a multicarrier system. The mobility spectrum analysis has been used extensively in multi-band semiconductors, in multi-band topological semi-metals [2] and more limited in multi-band iron-based superconductors [3, 4]. The conductivity of a multi-band system is given in terms of a mobility spectrum, based on a method described in Ref. 5 This work is part of a separate report related to the mobility spectrum of $FeSe_{1-x}S_x$ [6].

$$\hat{\sigma}(B) = \sigma_{xx} + i\sigma_{xy} = \int_{-\infty}^{\infty} d\mu \frac{s(\mu)(1 + i\mu B)}{1 + \mu^2 B^2} \tag{S10}$$

where $s(\mu) = e\mu n(\mu)$ is the mobility spectrum of zero-field conductivity and the charge carrier densities are functions of mobility (with electrons defined to have negative μ and $n(\mu)$). Thus the fundamental object of interest to identify the properties of charge carriers in a material becomes the conductivity spectrum $s(\mu)$, or equivalently the carrier-density spectrum $n(\mu)$. The mobility spectrum constructed for t=125 nm in Figure 2(a) is expanded in different components in Fig. S9. The initial parameters proposed by the mobility spectrum, together with compensation of the charge carriers, are used to extract the discrete fitted parameters in Figs. 2(d),(e) and (f). The behaviour of the mobilities and carrier densities as a function of temperature are consistent between the two approaches.

The mobility spectrum could be a powerful tool to identify the conduction in multiple band systems with different mobility which appear as distinct peaks in $s(\mu)$, as long as the condition $\mu B < 1$ is satisfied. Its fundamental description of the electrical

transport does not require advance knowledge about the band structure or the scattering mechanisms. A distribution of relaxation times results in a distribution of mobilities and broadening of the corresponding peak in $s(\mu)$. Therefore, the shape of the peak in $s(\mu)$ reveals not only an average of relaxation times ($\langle\tau\rangle^2/(\tau)^2$), but the actual distribution of relaxation times [5].

Approximations are often used to extract the form of the mobility spectrum for a material having a finite data set of conductivity or resistivity measurements as $s(\mu)$ is never fully constrained on the infinite set of basis functions. Discrete approximations can often add ghost peaks in the spectrum, as well as the introduction of bias with a necessary predetermined range of interest for mobility. Other techniques, used in the case of iron-based superconductors, fit analytic forms to the data which can be directly transformed [3, 4]. These approaches have the disadvantages of losing some fidelity of the data and require an analytic continuation of the fitted function to infinite magnetic field values.

The Lifshitz-Kosevich equation

The amplitude of quantum oscillations can be described by the Lifshitz-Kosevich equation [7, 8]:

$$\Omega = \left(\frac{e}{2\pi\hbar}\right)^{3/2} \frac{e\hbar V B^{5/2}}{m^*\pi^2} \sum_{A_{\text{ext}}} \left|\frac{\partial^2 A_{k,i}}{\partial k_\perp^2}\right|^{-1/2} \sum_{p=1}^{\infty} p^{-5/2} R_T R_{\text{D}} R_S, \cos\left(2\pi p\left(\frac{F}{B} - \frac{1}{2} + \frac{\phi_B}{2\pi}\right)\left[\pm\frac{\pi}{4}\right]\right), \tag{S11}$$

where R_T, R_{D}, and R_S are damping terms. The first sum extends over all extremal Fermi surface areas $A_{k,i}$ perpendicular to the applied field, while the second over all p harmonics of the fundamental oscillation frequency. These oscillations are periodic in $1/B$ with a frequency which is determined by orbits on the Fermi Surface that enclose locally extremal momentum space area, where $F_i = \frac{\hbar}{2\pi e} A_{k,i}$.

The first damping term, R_T, accounts for the thermal broadening from the Fermi-Dirac distribution with temperature and is given by:

$$R_T = \frac{X}{\sinh(X)} \qquad X = \frac{2\pi^2 k_B T \text{pm}^*}{e\hbar B} \tag{S12}$$

where m^* is the quasiparticle effective mass. R_T depends on the ratio $X \propto k_B T/\hbar\omega_c$, where $\omega_c = eB/m^*$ is the cyclotron frequency. These two equations contain the temperature dependence of the amplitude of oscillation, and by fitting data to Equation S12 the effect mass of m^* can be extracted.

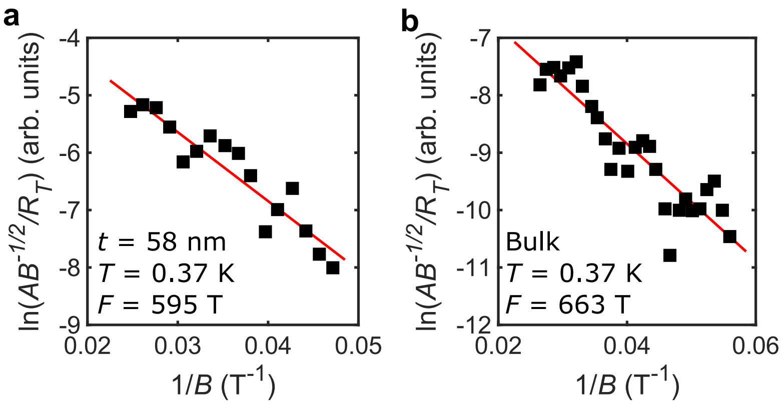

FIG. S2. **Estimation of the quantum scattering time from Dingle plots.** Dingle plots to extract the Dingle temperature, T_{D}, from the slope and accounting for the R_T and R_{D} field dependence for (a) a $t = 58$ nm device ($T_{\text{D}} \sim 4.5(4)$ K) and (b) a bulk single crystal S1 ($T_{\text{D}} \sim 1.6(2)$ K).

The second damping term, R_D, is accounts for the impurity scattering of the electrons and is given by

$$R_{\text{D}} = \exp\left(-\frac{\pi m_b}{eB\tau}\right) = \exp\left(-\frac{2\pi^2 m_b k_B T_{\text{D}}}{e\hbar B}\right), \tag{S13}$$

where τ is the scattering time, m_b is the band mass, and $T_{\text{D}} = \hbar/2\pi k_B\tau$ is the Dingle temperature. This equations is determined by the impurity scattering rate which acts to exponentially dampen the amplitude of the quantum oscillation in magnetic field.

As the band structure calculations that provide the band mass cannot capture the Fermi surface of FeSe correctly [9], we use $m^* = m_b$ to determine the Dingle temperature. The values of the bulk were taken from previous reports [10, 11]. The third damping term, R_S, accounts for the Zeeman splitting of Landau levels.

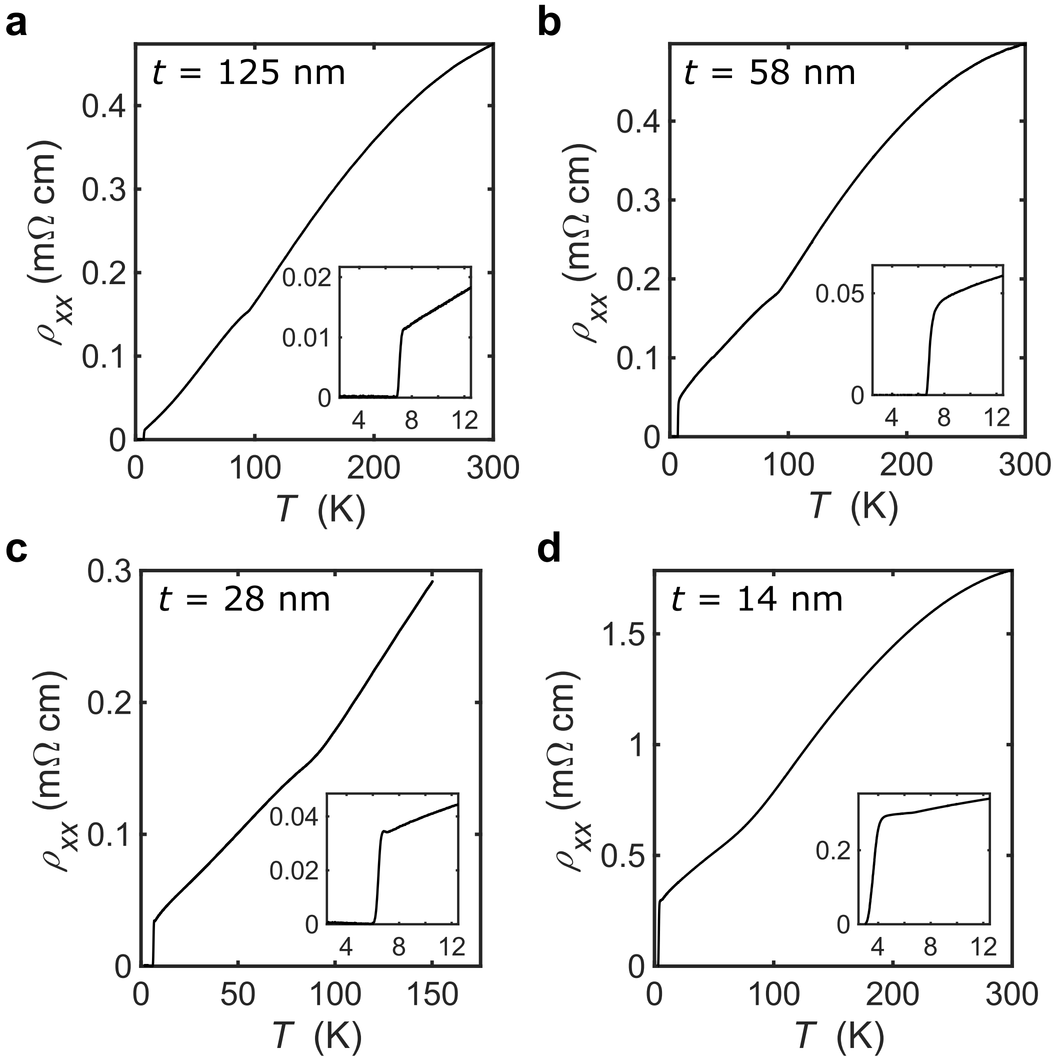

FIG. S3. **Resistivity in zero-magnetic field of thin flakes devices.** Temperature dependence of the zero-field resistivity for four thin flake devices with different thicknesses (a) t =125 nm, (b) t = 58 nm, (c) t = 28 nm and (d) t =14 nm. The insets show the low temperature superconducting transition region and highlights that at low temperature the resistivity has a rather linear dependence for most of the thin flakes samples. Additional resistivity curves for other thin flakes were previously reported in Ref. [1]. A small increase in resistivity of the thinnest flakes is detected in (c) and (d), which is often interpreted as a signature of Anderson localization due to disorder. However, insulating behaviour was detected in the ultra-thin limit of FeSe flakes below 9 nm [1, 12] and the number of charge carriers remain unchanged upon reducing thickness (see Fig. 2(d) and Ref. [12]).

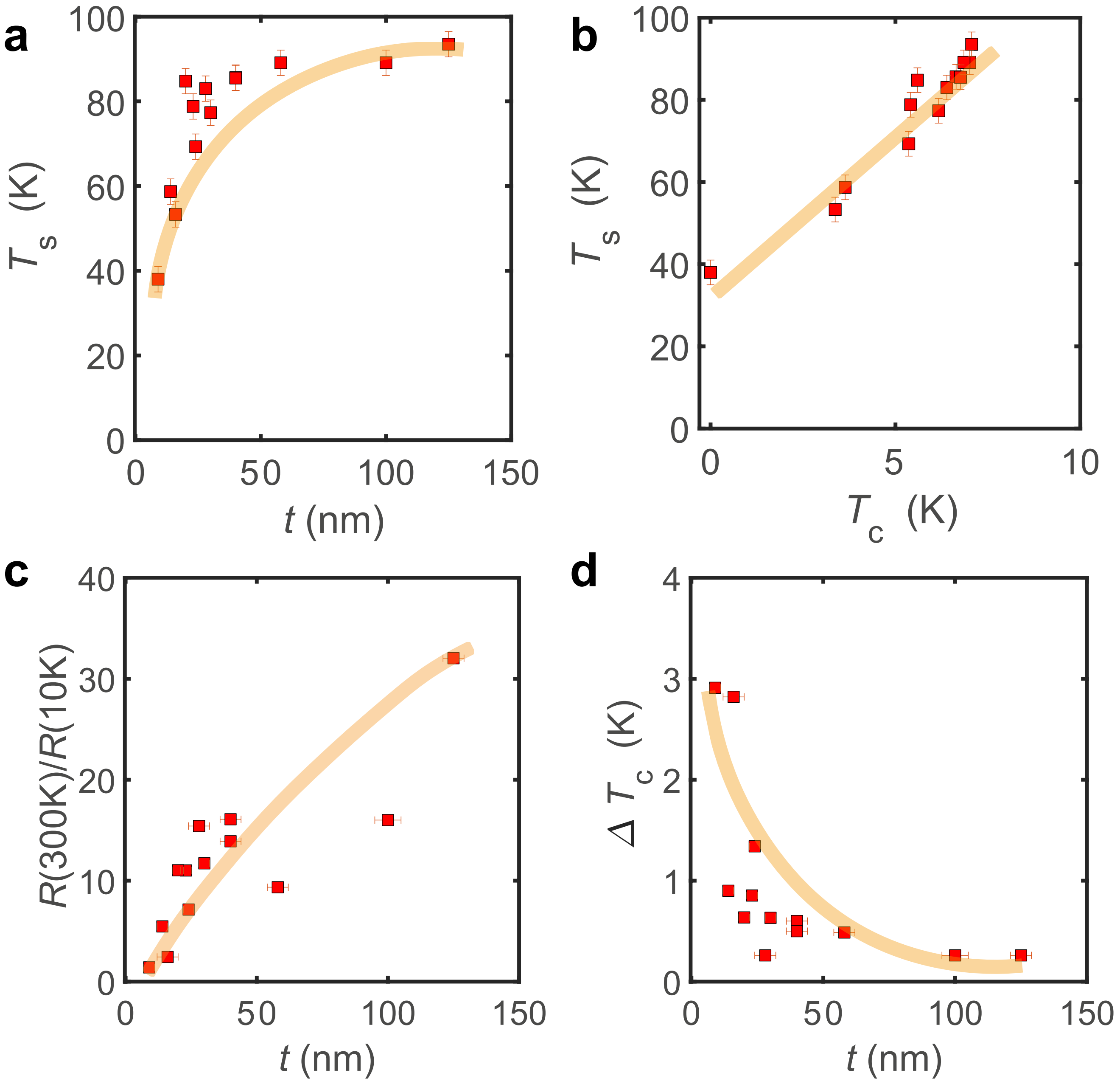

FIG. S4. **Transport parameters of FeSe thin flakes.** (a) Thickness dependence of the nematic transition T_s defined as the minimum in $d\rho_{xx}/dT$. The value of the transition of the bulk FeSe single crystals is T_s=89 K. (b) The linear relationship between T_s and the critical temperature, T_c, defined here as the middle of the superconducting transition. The value of the critical temperature of the bulk FeSe single crystals is always higher $T_c \sim 8.7$ K. (c) The thickness dependence of the residual resistivity ratio, defined as the ratio between the resistivity at 300 K and 10 K, $RRR = \rho(300\text{ K})/\rho(10\text{ K})$. The R for the bulk are is around 32 [13]. (d) The thickness dependence of the width of the superconducting transition, $\Delta T_c = T_{c,\text{on}} - T_{c,\text{off}}$. The solid lines in all panels are guides to the eye.

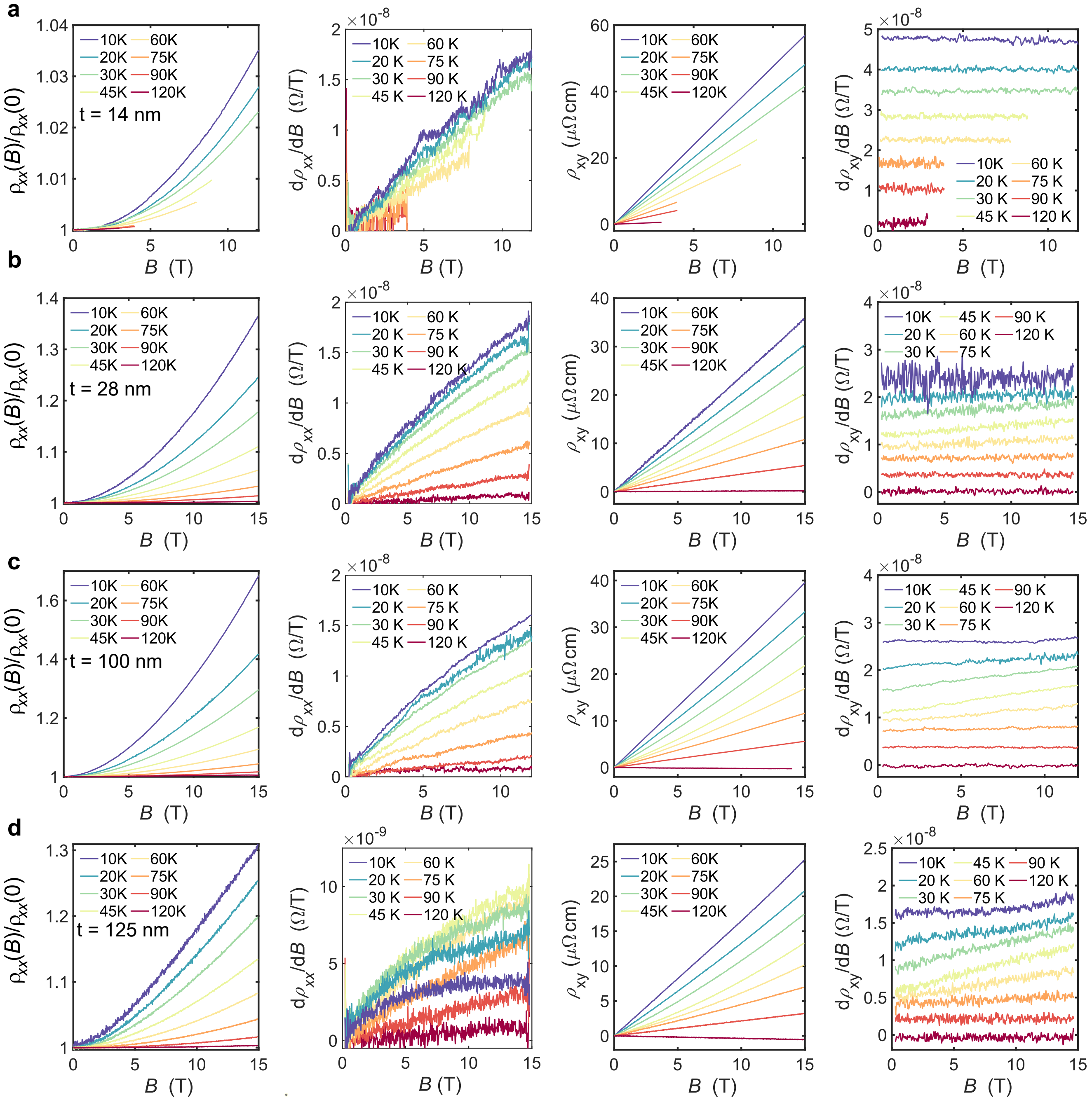

FIG. S5. **The magnetotransport data of different thin flake devices.** The field dependence of the longitudinal magnetoresistance, ρ_{xx}, and its derivative, $d\rho_{xx}/dB$, the Hall component, ρ_{xy}, and its derivative $d\rho_{xy}/dB$ for different thin flake devices with thicknesses (a) $t=$14 nm, (b) $t=$28 nm, (c) $t=$100 nm and (d) $t=$125 nm measured at different constant temperatures.

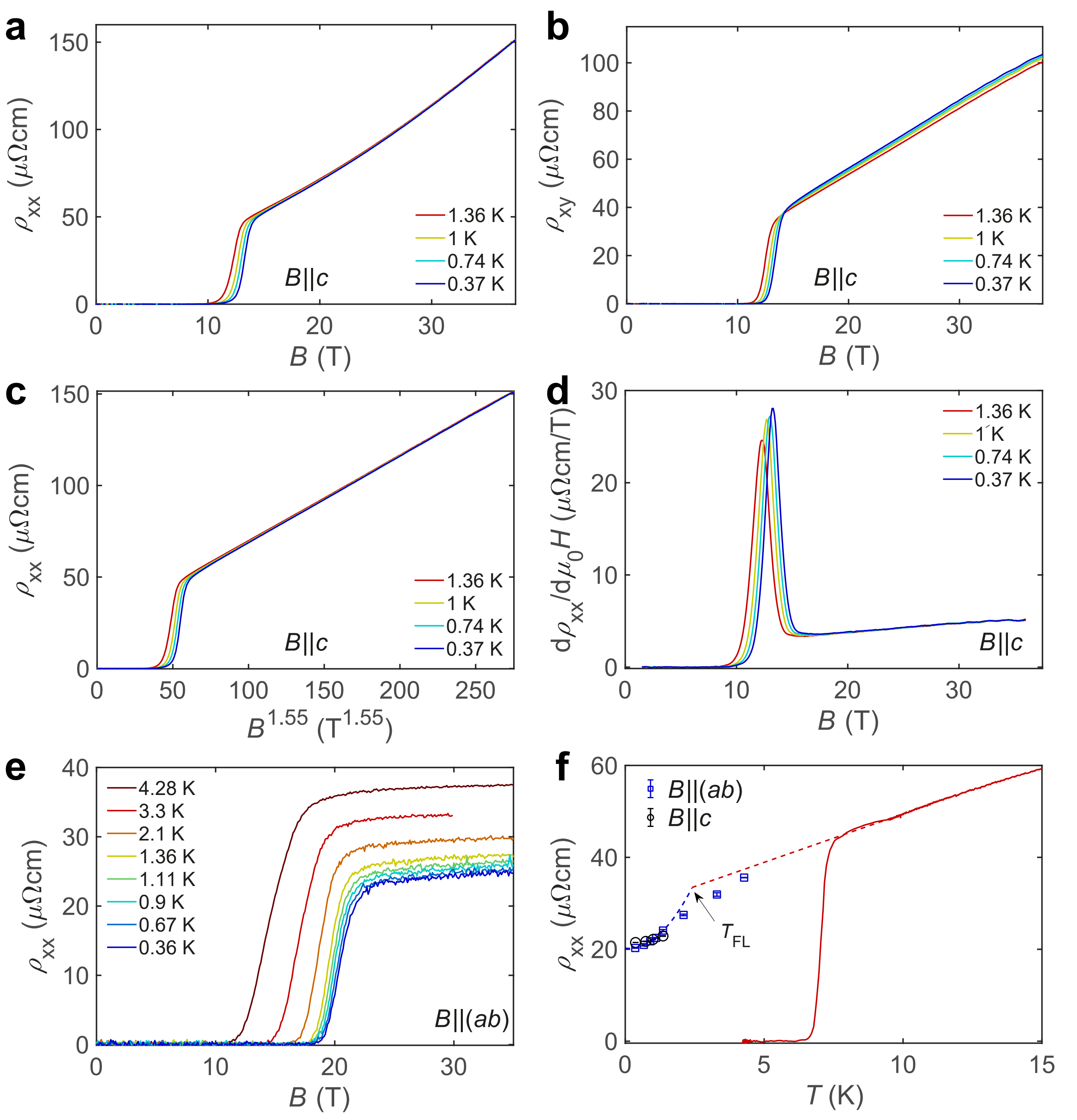

FIG. S6. **High magnetic field study of a t =58 nm device.** High magnetic field dependence of (a) the longitudinal magnetoresistance, ρ_{xx} and (b) the transverse Hall component, ρ_{xy}, for a device with t =58 nm measured at different constant temperatures. (c) The longitudinal resistance versus $B^{1.6}$ shows a linear dependence, similar to bulk single crystals [13]. (d) The derivative of the longitudinal resistance, $d\rho_{xx}/dB$. (e) The in-plane resistance versus magnetic field for different temperatures. (f) The low-temperature dependence of resistance. The extrapolated values in zero field were extracted from (e) and (c). The dashed line is a linear fit to the high temperature data that follows the extracted data to the lowest temperature.

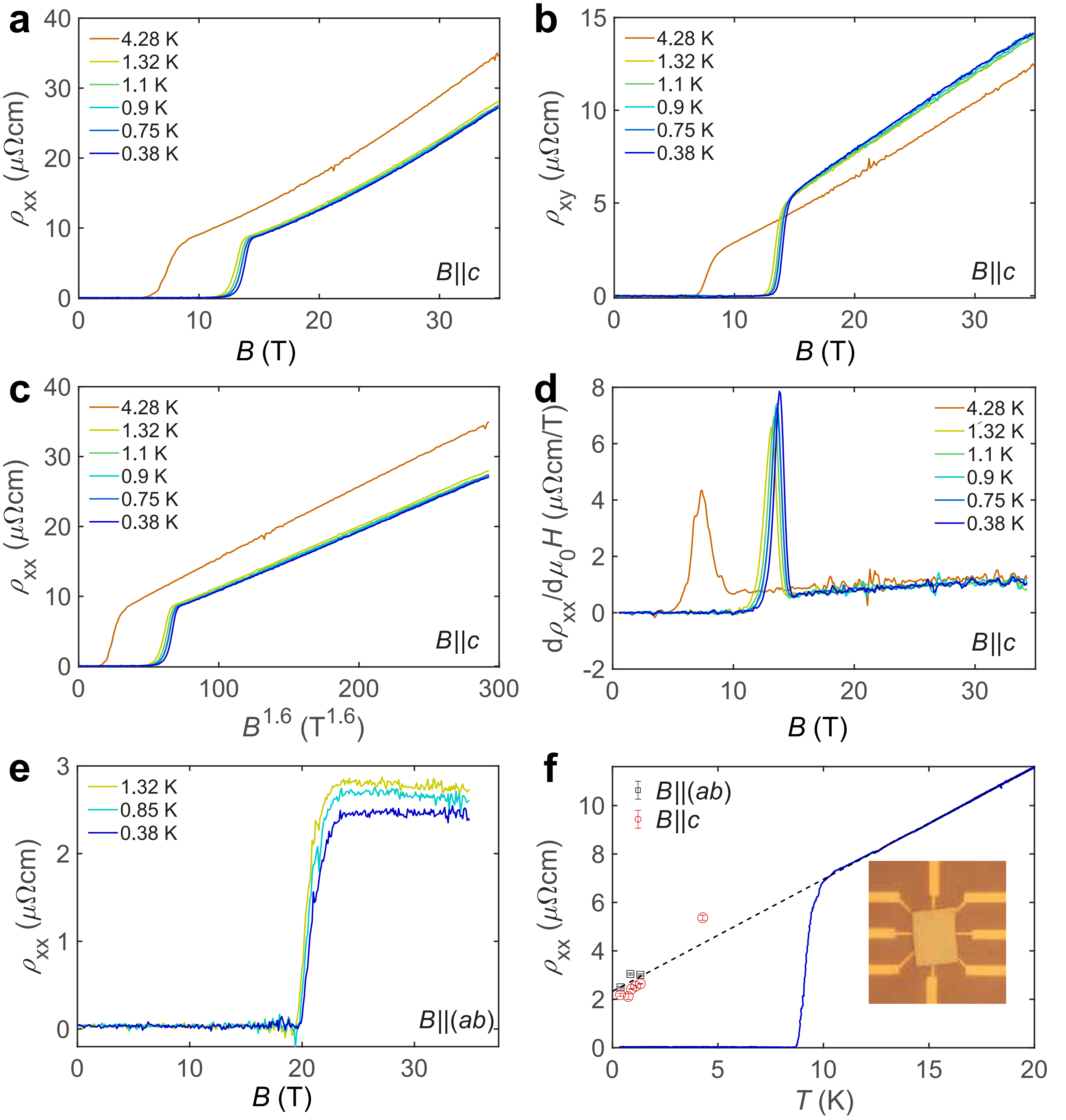

FIG. S7. **High magnetic field study of a t=100 nm device (LF8a).** High magnetic field dependence of (a) the longitudinal magnetoresistance, ρ_{xx} and (b) the transverse Hall component, ρ_{xy}, for a device with t=100 nm measured at different constant temperatures. (c) The longitudinal resistance versus $B^{1.6}$ show a linear dependence, similar to bulk single crystals [13]. (d) The derivative of the longitudinal resistance, $d\rho_{xx}/dB$. (e) The in-plane resistance versus magnetic field for different temperatures. (f) The low-temperature dependence of resistance. The extrapolated values in zero field were extracted from (e) and (c). The dashed line is a linear fit to the high temperature data that follows the extracted data to the lowest temperature.

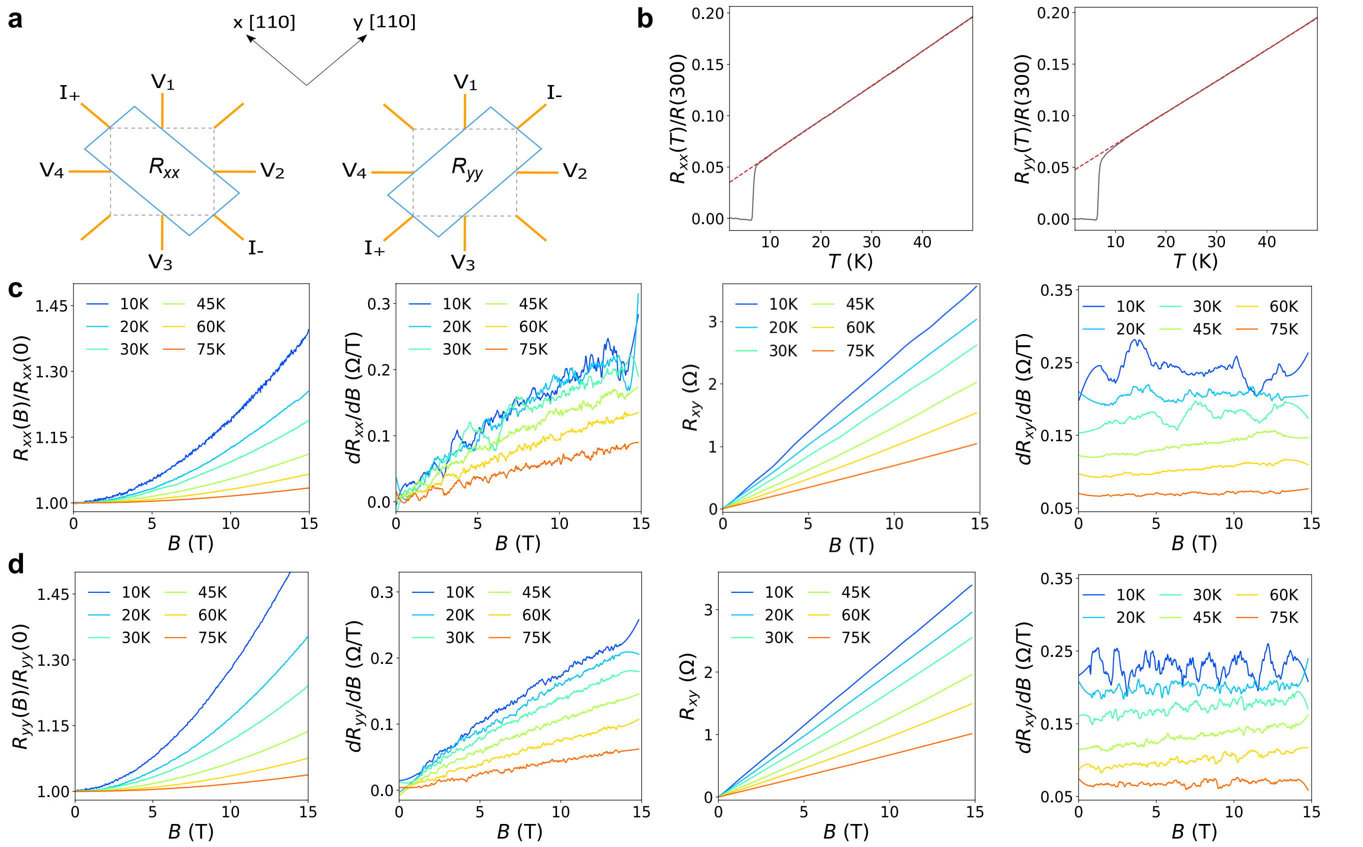

FIG. S8. **Anisotropy of a multi-contact thick device** $t = 100$ **nm.** (a) The different configurations used to probe the in-plane anisotropy. (b) The temperature dependence of the R_{xx} and R_{yy} components. (c) The field dependence of the longitudinal magnetoresistance, R_{xx} and the transverse Hall component, R_{xy} together with their corresponding derivatives. (d) The longitudinal and transverse components for the second configuration for the symmetric longitudinal component, R_{yy}, and the antisymmetric transverse component, R_{xy} and their derivatives.

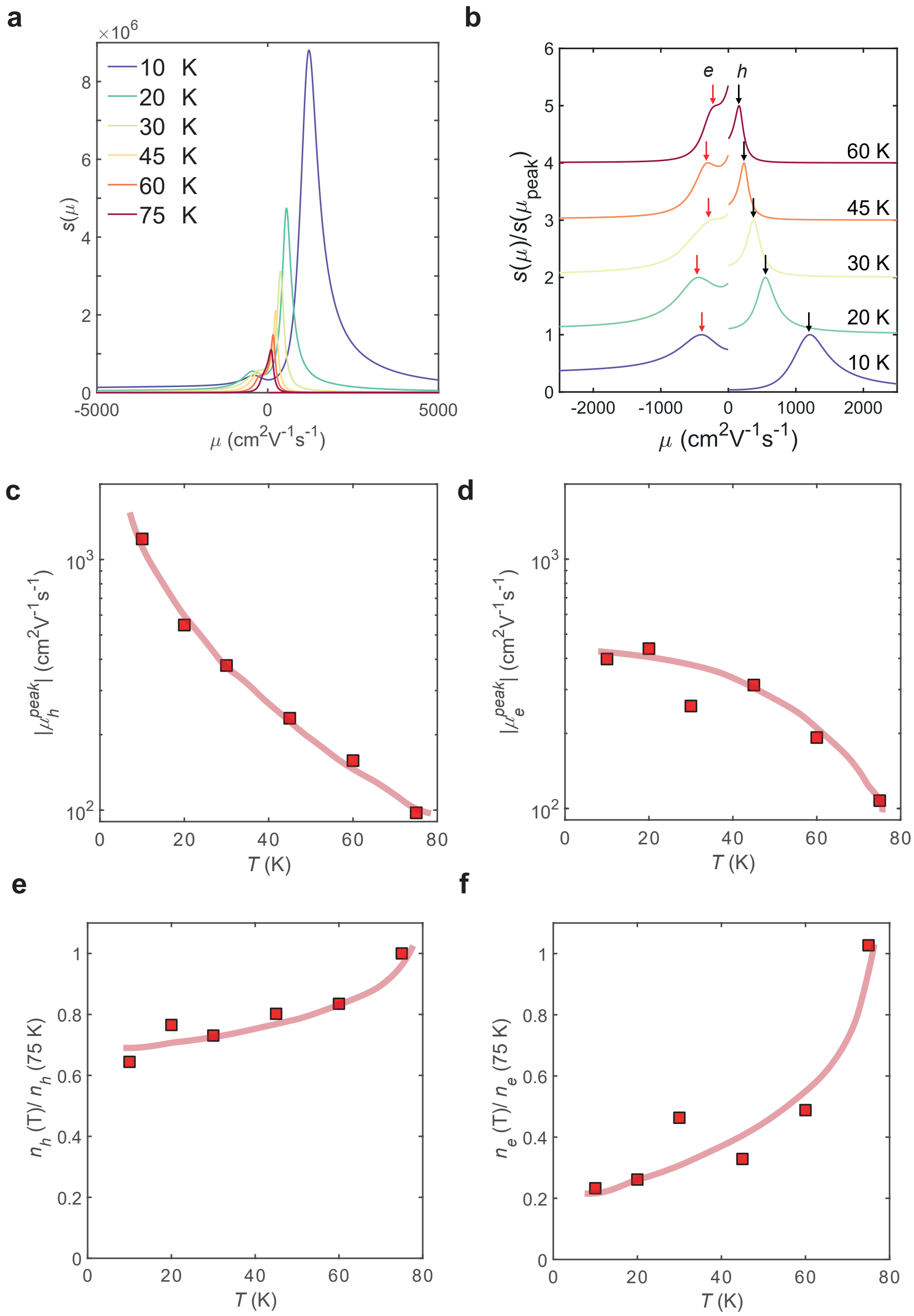

FIG. S9. **The mobility spectrum of the thick device $t = 125$ nm.** (a) The raw mobility spectrum, $s(\mu) = e\mu n(\mu)$ constructed based on the raw data from Fig. 1(c) and (d). (b) The mobility spectrum divided to mobility value at the maximum peak at each temperature, $s(\mu)/e\mu$. (c) and (d) The temperature dependence of the mobilities of positive (μ_h) and negative charge (μ_e) carriers extracted from the peak position from (b). (e) and (f) The temperature dependence of the number of charge carriers, n_h and n_e extracted from the corresponding peak positions $n(\mu)$=$s(\mu)/(e \cdot \mu)$ from (a) and normalized to the value at 75 K. The solid lines are guide to the eye. The normalized value of the carrier densities in (e) and (f) are affected by finite field points and other scattering effects that cause broadening of the peaks [5].

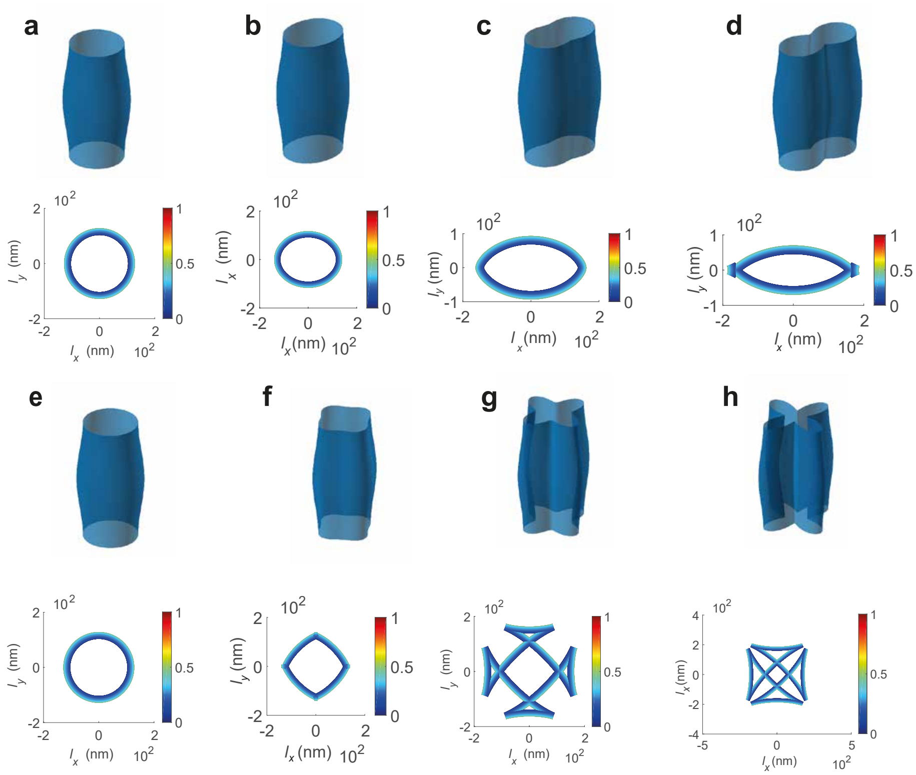

FIG. S10. **Fermi surfaces and scattering path length.** (top panels) The evolution of the Fermi surface and the scattering length for a quasi-two dimensional Fermi surface expanded in cylindrical coordinates [14] with $k_{00} = 0.1$ Å^{-1}, the interplane parameter of $k_{10} = 0.01$ Å^{-1}, and the in-plane two-fold symmetric parameter (a) k_{02}=0, (b) k_{02}=0.01, (c) k_{02}=0.03, (d) k_{02}=0.05 Å^{-1}. (bottom panels) The evolution of the Fermi surface and the scattering length of the in-plane four-fold symmetric parameter (e) k_{04}=0, (f) k_{04}=0.01, (g) k_{04}=0.03, (h) k_{04}=0.05 Å^{-1}. The Hall conductivity is linked to the $2 \times A_\ell$, area swept by the scattering path length, $\ell_{\mathbf{k}}$. In the cases (g) and (h) the Hall conductivity can change sign as compared with the other cases. The scattering time is assumed isotropic, $\tau = 1$ ps [15].

[1] L. S. Farrar, M. Bristow, A. A. Haghighirad, A. McCollam, S. J. Bending, and A. I. Coldea, Suppression of superconductivity and enhanced critical field anisotropy in thin flakes of FeSe, npj Quantum Materials **5**, 29 (2020).

[2] H. Zhao, W. Li, Y. Chen, C. Xu, B. Li, W. Luo, D. Qian, and Z. Shi, Transport property of multi-band topological material $PtBi_2$ studied by maximum entropy mobility spectrum analysis (MEMSA) , Scientific Reports **11**, 6249 (2021).

[3] K. K. Huynh, Y. Tanabe, T. Urata, H. Oguro, S. Heguri, K. Watanabe, and K. Tanigaki, Electric transport of a single-crystal iron chalcogenide FeSe superconductor: Evidence of symmetry-breakdown nematicity and additional ultrafast Dirac cone-like carriers, Phys. Rev. B **90**, 144516 (2014).

[4] K. K. Huynh, Y. Tanabe, T. Urata, H. Oguro, S. Heguri, K. Watanabe, and K. Tanigaki, Electric transport of a single-crystal iron chalcogenide FeSe superconductor: Evidence of symmetry-breakdown nematicity and additional ultrafast Dirac cone-like carriers, Phys. Rev. B **90**, 144516 (2014).

[5] W. A. Beck and J. R. Anderson, Determination of electrical transport properties using a novel magnetic field-dependent hall technique, Journal of Applied Physics **62**, 541 (1987), https://doi.org/10.1063/1.339780.

[6] O. Humphries, Mobility spectrum analysis for multiband superconducting systems, MPhys project, University of Oxford (2016).

[7] I. Lifshitz and L. Kosevich, On the theory of the Shubnikov-de Haas effect, Sov. Phys. JETP **6**, 67 (1958).

[8] D. Shoenberg, *Magnetic Oscillations in Metals* (Cambridge University Press, Cambridge, 1984).

[9] M. D. Watson, S. Backes, A. A. Haghighirad, M. Hoesch, T. K. Kim, A. I. Coldea, and R. Valentí, Formation of Hubbard-like bands as a fingerprint of strong electron-electron interactions in FeSe, Phys. Rev. B **95**, 081106 (2017).

[10] M. D. Watson, T. K. Kim, A. A. Haghighirad, N. R. Davies, A. McCollam, A. Narayanan, S. F. Blake, Y. L. Chen, S. Ghannadzadeh, A. J. Schofield, M. Hoesch, C. Meingast, T. Wolf, and A. I. Coldea, Emergence of the nematic electronic state in FeSe, Phys. Rev. B **91**, 155106 (2015).

[11] A. I. Coldea, S. F. Blake, S. Kasahara, A. A. Haghighirad, M. D. Watson, W. Knafo, E. S. Choi, A. McCollam, P. Reiss, T. Yamashita, M. Bruma, S. Speller, Y. Matsuda, T. Wolf, T. Shibauchi, and A. J. Schofield, Evolution of the low-temperature Fermi surface of superconducting $FeSe_{1-x}S_x$ across a nematic phase transition, npj Quantum Materials **4**, 2 (2019).

[12] C. S. Zhu, B. Lei, Z. L. Sun, J. H. Cui, M. Z. Shi, W. Z. Zhuo, X. G. Luo, and X. H. Chen, Evolution of transport properties in FeSe thin flakes with thickness approaching the two-dimensional limit, Phys. Rev. B **104**, 024509 (2021).

[13] M. Bristow, P. Reiss, A. A. Haghighirad, Z. Zajicek, S. J. Singh, T. Wolf, D. Graf, W. Knafo, A. McCollam, and A. I. Coldea, Anomalous high-magnetic field electronic state of the nematic superconductors $FeSe_{1-x}S_x$, Phys. Rev. Research **2**, 013309 (2020).

[14] J. C. A. Prentice and A. I. Coldea, Modeling the angle-dependent magnetoresistance oscillations of fermi surfaces with hexagonal symmetry, Phys. Rev. B **93**, 245105 (2016).

[15] N. P. Ong, Geometric interpretation of the weak-field Hall conductivity in two-dimensional metals with arbitrary Fermi surface, Phys. Rev. B **43**, 193 (1991).
